# Association Between Triglyceride–Glucose Index and Risk of Cancer: A Systematic Review and Meta-Analysis

**DOI:** 10.3390/jpm16050274

**Published:** 2026-05-20

**Authors:** Roberto Fabiani, Valentina Squadroni, Patrizia Rosignoli

**Affiliations:** Department of Chemistry, Biology and Biotechnology, University of Perugia, 06123 Perugia, Italy; valentina.squadroni@studenti.unipg.it (V.S.); patrizia.rosignoli@unipg.it (P.R.)

**Keywords:** triglyceride–glucose index, TyG index, insulin resistance, cancer risk, meta-analysis, systematic review

## Abstract

**Background/Objectives**: The triglyceride–glucose (TyG) index, a reliable marker for insulin resistance, is strongly associated with T2DM, hypertension, and cardiovascular disease. Less well known is its relationship with cancer risk. The aim of this study was to quantify the association between the TyG index and risk of different types of cancer. **Methods**: Publications were searched in the PubMed, Web of Science, and Scopus databases using appropriate keywords. The PICOS framework was used to select the studies, and their quality was evaluated according to the “Newcastle–Ottawa Scale” (NOS). Meta-analysis was performed through a random-effects model using cancer risk parameters (RR: relative risk, OR: odds ratio and HR: hazard ratio) extracted from 26 selected studies associated with TyG index values. The weighted mean difference (WMD) was used to compare the mean of the TyG index in cancer patients to that of the control group. Heterogeneity was assessed by Cochran’s Q and I^2^ statistics, while publication bias was evidenced using the Egger test and the Begg test, and funnel plot asymmetry. **Results**: A higher TyG index value was observed in cancer subjects (9483) compared to healthy controls (978,675) (WMD: 0.23, 95% CI: 0.16–0.31, *p* < 0.0001, n = 15). A statistically significant increase in cancer risk was associated with the TyG index level, expressed as both a categorical (OR 1.33, 95% CI 1.22–1.45, *p* < 0.0001, n = 29) and continuous (OR 1.14, 95% CI 1.10–1.19, *p* < 0.0001, n = 27) variable. The effect was more evident in case–control/cross-sectional studies compared to cohort studies (OR 1.78, 95% CI 1.51–2.09 vs. OR 1.19, 95% CI 1.10–1.29 TyG categorical; OR 1.46, 95% CI 1.21–1.76 vs. OR 1.09, 95% CI 1.05–1.12 TyG continuous). Stratified analysis showed an increased risk of cancer occurrence for gastrointestinal, gynecological, colorectal, breast, and gastric sites, while no association was observed for endometrial, ovarian, prostate, lung or esophageal cancers. **Conclusions**: Our results evidence an increase in cancer risk associated with higher TyG index values. However, due to the low number of studies, the effect on specific tumor sites was not statistically significant. Additional epidemiological studies with a cohort design are necessary to confirm these associations.

## 1. Introduction

Despite significant advances in diagnosis and treatment, cancer continues to represent a major social, economic and public health problem worldwide. According to the latest data from the Global Cancer Observatory (GLOBOCAN), nearly 20 million new cases of cancer were diagnosed worldwide in 2022, resulting in 9.7 million deaths [1], and it is a condition that is expected to worsen over time, with an estimated incidence that may reach 35 million new cases in 2050 [1]. Identifying risk factors and implementing interventions to reduce population exposure to them are crucial strategies for preventing and mitigating cancer risk. It is currently estimated that approximately 44% of all cancer deaths are attributable to largely modifiable behavioral, environmental, and occupational risk factors [2,3]. Although several cancer risk factors, such as tobacco smoking, alcohol intake, air pollution and obesity, have been well established and their tumor initiation/promotion mechanisms elucidated [3], others are undergoing extensive investigation. In particular, the growing prevalence of metabolic disorders—including dyslipidemia, hyperglycemia and insulin resistance (IR)—has prompted further investigation into their roles in cancer etiology [4].

IR is a pathological metabolic condition characterized by a reduced responsiveness of peripheral tissues (muscle, liver, and adipose) to the metabolic actions of insulin, particularly its ability to promote glucose uptake and inhibit hepatic gluconeogenesis [5]. IR is a central feature of metabolic syndrome and is strongly associated with the development of type 2 diabetes mellitus (T2DM), atherosclerosis, and non-alcoholic fatty liver disease [5]. Although the mechanisms involved in the development of IR resistance have not been fully elucidated, IR-related conditions have been implicated in carcinogenesis through several biological pathways [6]. Chronic hyperinsulinemia may exert mitogenic effects by activating insulin-like growth factor-1 (IGF-1) signaling, promoting cellular proliferation and survival while inhibiting apoptosis [6]. Additionally, the pro-inflammatory and pro-oxidative state accompanying IR may contribute to tumorigenesis by enhancing DNA damage, angiogenesis, and metastatic potential [6].

Numerous epidemiological studies have demonstrated associations between IR and an increased risk of cancers, particularly breast, colorectal and endometrial cancers [7,8,9]. However, direct evaluation of IR in clinical practice is limited by the complexity and cost of standard methods used to measure it, such as the hyperinsulinemic–euglycemic clamp test and the homeostasis model assessment for IR (HOMA-IR) [10]. Recently, the triglyceride–glucose (TyG) index has emerged as a reliable, low-cost, and accessible surrogate marker for IR. It is derived from fasting triglyceride and glucose levels, and it is generally calculated using the following formula: TyG index = ln [fasting triglycerides (mg/dL) × fasting glucose (mg/dL)/2] [11]. The TyG index is a cost-effective and reproducible parameter easily applicable in both research and clinical settings. It has shown strong correlations with HOMA-IR and even with gold-standard clamp studies, making it a valid alternative for assessing IR in large population studies [12]. Moreover, it has been associated with a variety of IR-related conditions, such as T2DM, hypertension, and cardiovascular disease [13,14,15].

Although a wide body of evidence exists linking the TyG index to multiple health outcomes, its role in cancer risk remains relatively underexplored [16,17]. Some studies have reported positive associations between a higher TyG index and increased risk of certain cancers, including colorectal, liver, and breast cancer, while others have found no significant correlation. Preliminary results have been summarized in a 2023 meta-analysis of six observational studies, including over 992,000 participants, which found that individuals with a higher TyG index had a 14% increased risk of cancer (RR = 1.14, 95% CI: 1.08–1.20) compared to those with lower TyG levels [18]. Since this meta-analysis was published, a multitude of studies have appeared in the literature reporting the association between the TyG index and the occurrence of cancer at different sites, with contrasting results. Therefore, a comprehensive synthesis of the existing literature is warranted to clarify the relationship between the TyG index and cancer risk.

The primary objective of this study was to conduct a systematic review and meta-analysis to evaluate the association between the TyG index and the appearance of cancer. Secondary objectives include exploring potential effect modifiers such as cancer types and study design.

## 2. Materials and Methods

This systematic review and meta-analysis was performed according to the MOOSE (Meta-analysis Of Observational Studies in Epidemiology) guidelines and the PRISMA (Preferred Reporting Items for Systematic Reviews and Meta-Analyses) statement (File S1) [19,20]. The study protocol has been registered in the International Prospective Register of Systematic Reviews (www.crd.york.ac.uk/PROSPERO/ Registration No: CRD420251008292) (accessed on 26 May 2025). The PRISMA checklist can be seen with Supplementary Materials in File S1.

A literature search was carried out without restrictions (neither of language nor of time) on the following databases: PubMed (http://www.ncbi.nlm.nih.gov/pubmed/, accessed on 6 March 2025), Web of Science (http://wokinfo.com/, accessed on 6 March 2025) and Scopus (https://www.scopus.com/, accessed on 6 March 2025). The eligibility of studies was decided according to the PICOS framework. (P) population: healthy adult subjects or adult patients with cancer in any site; (I) intervention/exposure: measured TyG index and calculated as above reported using the fasting triglycerides (mg/dL) and glucose (mg/dL) concentrations [11]; (C) comparison: higher TyG index versus lower TyG index when it was analyzed as a categorical variable, or per 1-unit increase in when TyG index was analyzed as a continuous variable; (O) outcome: the prevalence or the incidence of cancer in any site; (S) study design: observational studies, such as case–control, cross-sectional, or cohort studies. Articles to be included in the systematic review were identified using a combination of the following Medical Subject Headings (MeSH) terms and keywords: (“Triglyceride glucose index” OR “TyG index”) AND (cancer OR neoplasm OR tumor OR tumour OR carcinoma OR adenocarcinoma OR adenoma OR neoplasia OR “neoplastic disease” OR malignancy). In order to find possible additional publications of interest, a reference list of included articles and recent relevant reviews was also examined. We excluded from the systematic review articles such as reviews; comments; editorials, meta-analyses; in vitro and animal studies; and studies that did not involve patients diagnosed with cancer, did not assess the TyG index as an exposure variable, or did not report multivariate-adjusted cancer risk estimates (RR, OR, or HR) with 95% confidence intervals (CI) across TyG index categories/increments. In cases where the same population had been examined in duplicate studies, the study with the largest number of subjects was incorporated in the meta-analysis. Initially, the selection of articles was performed considering the title and abstract, and then, the main text was evaluated for all studies that were not discarded in the first phase. Two authors (R.F. and V.S.) independently performed the study selection. Discrepancies were resolved by discussion with a third author (P.R.). The list of selected studies, the removal of duplicates, and the selection of studies of interest were managed with Zotero 9.

Two authors (R.F. and V.S.) independently extracted from each selected article the following information: first author, year of publication, location, study design and name, population characteristics (number of cases and controls, incident cases, length of follow-up, age), TyG index measurement/calculation method and values, type of cancer, TyG parametrization, OR/RR/HR (95% CI), p for trend, matched or adjusted variables, and quality of the study (NOS scores). When multiple estimates were reported in the article, those adjusted for the most confounding factors were extracted. The quality evaluation of the selected studies was performed according to the “Newcastle–Ottawa Scale” (NOS) [21]. The NOS uses a star system, with a total score ranging from 0 to 9. An NOS score equal to or superior to 7 indicated a high-quality study. The quality assessment of each selected study was carried out individually by two researchers, and disagreements were resolved by a joint re-evaluation of the original article with a third author.

The weighted mean difference (WMD) was used to compare the mean (SD) of the TyG index in patients with cancer to that of the control group. The WMD and the association between the TyG index and cancer risk were assessed using the statistical program ProMeta version 3.0 (IDo Statistics-Internovi, Cesena, Italy). The relative risk and hazard ratio were taken as an approximation to the OR, and the meta-analysis was performed as if all types of ratios were ORs. When the study divided the TyG into tertiles, quartiles, and quintiles, the combined risk estimates were calculated considering the first quantile as the reference (lower TyG index) and the exposure as the last quantile (higher TyG index). When data on TyG were presented as continuous variables, the combined risk estimates were calculated per 1-unit increment. In both cases, the risk was calculated using a random effect model. Heterogeneity between studies was evaluated by the chi-square-based Cochran’s Q statistic and the I^2^ statistic and was considered significant if *p* < 0.05 or I^2^ > 50% [22,23]. Publication bias was detected by Begg’s and Egger’s tests [24,25]. Both methods were tested for funnel plot asymmetry—the former was based on the rank correlation between the effect estimates and their sampling variances, and the latter was based on a linear regression of a standard normal deviate on its precision. If a potential bias was detected, we further conducted the “trim and fill” funnel plot-based method of testing and adjusting for publication bias. We also conducted a sensitivity analysis to investigate the influence of a single study on the overall risk estimate by omitting one study in each turn. We considered the funnel plot to be asymmetrical if the intercept of Egger’s regression line deviated from zero, with a *p*-value < 0.05.

## 3. Results

### 3.1. Studies Selection

**Figure 1:**
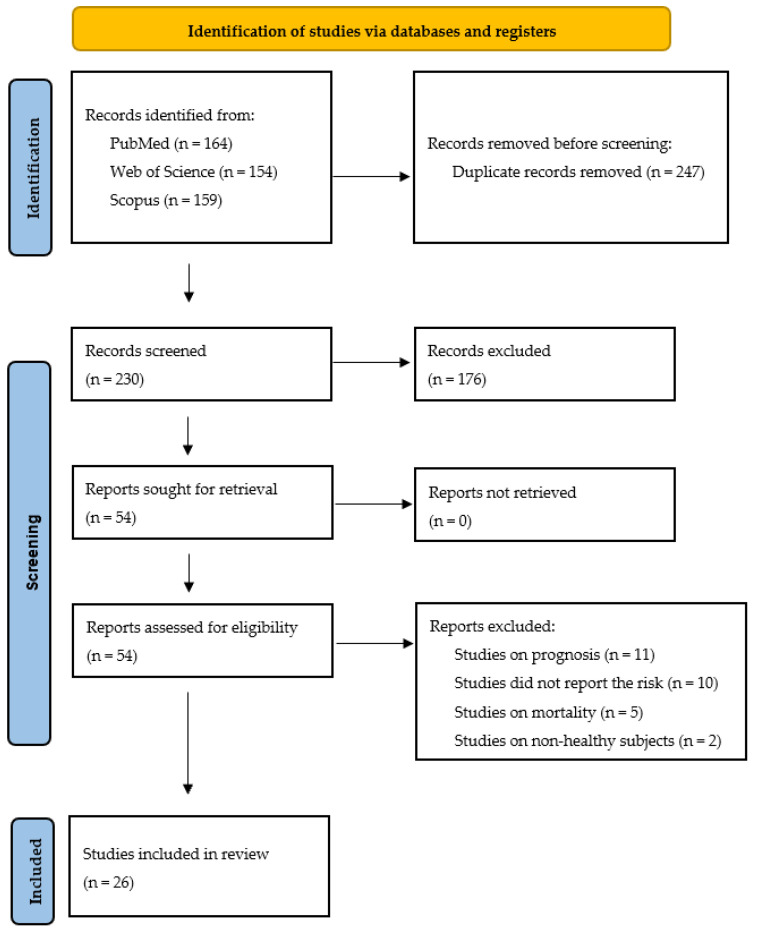
PRISMA flow chart illustrating the study selection process for the meta-analysis on TyG index and oncological risk.

From the initial search on three different databases, we selected a total of n = 477 articles (Figure 1). After removal of duplicates (n = 247), 230 items remained for selection on the basis of title and abstract analysis. Among them, 177 records were excluded because they were not appropriate for our systematic review. Therefore, 53 articles were selected for inclusion. One additional paper was identified from the bibliography lists of already selected articles, so 54 manuscripts were ultimately included for full-text analysis. Twenty-eight items were excluded because they did not meet the inclusion criteria. Specifically, 11 studies reported data only on cancer prognosis, 10 studies did not report the cancer risk estimates, five studies reported data on cancer mortality, and two studies reported data on non-healthy subjects. In the end, 26 articles [26,27,28,29,30,31,32,33,34,35,36,37,38,39,40,41,42,43,44,45,46,47,48,49,50,51] were selected for inclusion in the systematic review and meta-analysis (Figure 1).

### 3.2. Study Characteristics and Quality Assessment

Table 1 reports the main characteristics of the studies included in the systematic review. All studies were published from 2020 to 2024. Of the 26 selected studies; 13 were conducted in China [30,31,32,35,37,38,40,41,42,43,44,50,51]; four in Europe [26,29,39,47]; four in Korea [33,45,48,49]; two in China on a US population [36,46]; and one each in Turkey [34], Japan [27] and Indonesia [28]. Twelve were cohort studies in which the TyG index was measured before the cancer diagnosis [26,27,29,32,33,35,39,41,45,47,48,51]. Of these, 10 were prospective studies, and two were retrospective [33,45]. On the other hand, in nine cross-sectional studies [31,34,36,38,40,43,44,46,49] and in five case–control studies [28,30,37,42,50], the TyG index was measured post-diagnosis. All but one study measured the TyG index as ln, while the study by Alkurt et al. [34] did not. Four studies did not report the TyG value of the population sample [32,46,50,51], and six studies reported the TyG value for the entire population sample [26,35,36,39,45,48], while in the remaining sixteen studies, the TyG index was reported separately in cases and controls [27,28,29,30,31,33,34,37,38,40,41,42,43,44,47,49]. Fifteen studies reported data on gastrointestinal tumors [26,27,29,31,32,33,35,39,41,44,45,46,47,48,51], six studies reported data on breast cancer [26,28,36,40,43,51], and three each reported data on gynecological [26,36,42] and prostate cancers [37,38,39]. Nine studies reported cancer risk values associated with the TyG index analyzed as a categorical variable [28,33,34,37,38,43,45,46] and seven as a continuous variable [27,29,30,42,48,49,50], and eleven studies reported risk data as both a categorical and continuous variable [26,29,31,32,35,36,39,40,41,44,47,51]. The score for each domain of all studies included in the systematic review is shown in the Supplementary Materials (Table S1 for case–control/cross-sectional studies and Table S2 for cohort studies). The rightmost column of Table 1 shows the results of the quality assessment of each study. The NOS scores ranged from 7 to 9 (median: 8, mean ± SD: 8.15 ± 0.76). Six studies reported a score of 7 [32,35,41,42,44,51], ten studies reported a score of 8 [28,31,34,36,37,38,43,46,49,50], and ten studies reported a score of 9 [40].

### 3.3. Meta-Analysis on WMD

**Figure 2:**
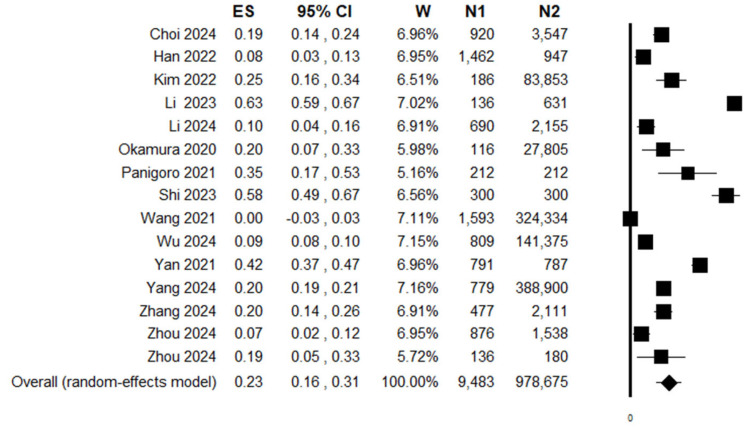
Forest plot showing the effect size (weighted mean difference: WMD) obtained comparing mean TyG index levels between cancer patients (N1) and healthy controls (N2) [27,28,29,30,31,33,37,38,40,41,42,43,44,47,49].

Fifteen studies reported the values of the TyG index separately in patients diagnosed with cancer compared to healthy controls. Among them, eleven studies, reporting data as mean ± standard deviations, were directly selected to calculate the WMD of the TyG index [27,29,30,31,33,38,41,42,43,44,49]. Four additional studies [28,37,40,47], reporting data as mean and 95% CI, were used to calculate the standard deviation and included in the WMD calculation. The analysis revealed statistically significantly higher levels of TyG index in cancer subjects (9483 cases) compared to healthy controls (978,675 subjects) (WMD: 0.23, 95% CI: 0.16–0.31, *p* < 0.0001, n = 15) (Figure 2). The heterogeneity was rather high (I2 = 97%), but sensitivity analysis showed that the WMD in the TyG level remained significant and varied from a value of 0.20 (95% CI: 0.14–0.26) excluding Li et al. 2023 [37] to a value of 0.25 (95% CI: 0.17–0.33), excluding Wang et al. 2021 [29] (Supplementary Figure S1). Stratifying the analysis according to the study design did not significantly reduced heterogeneity and produced the following results: case–control/cross-sectional studies (WMD: 0.28, 95% CI: 0.14–0.42, *p* < 0.0001, n = 10, I2 = 99%) and cohort studies (WMD: 0.14, 95% CI: 0.03–0.25, *p* = 0.015, n = 5, I2 = 98%).

### 3.4. Meta-Analysis on Cancer Risk

**Figure 3:**
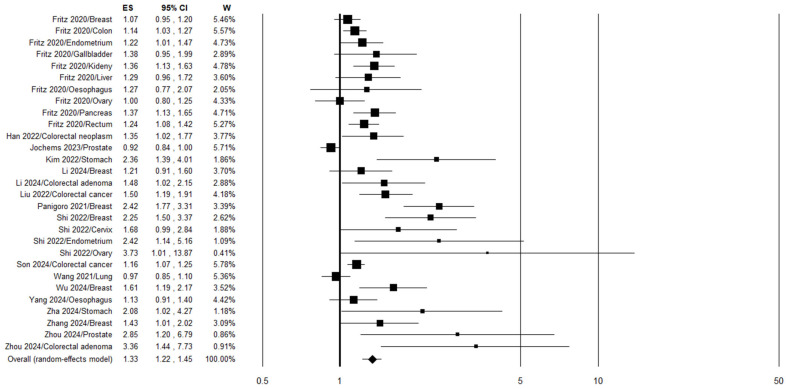
Forest plot for the meta-analyses regarding the association between the TyG index, analyzed as a categorical variable, and cancer risk [26,28,29,31,33,35,36,38,39,40,41,43,44,45,46,47,51].

Two articles were excluded from the meta-analysis on cancer risk [32,37] because they reported partial data that were later reported in subsequently published studies [35,38]. Another article was excluded since the TyG index was not calculated as ln [34]. The pooled results of the seventeen studies on the TyG index analyzed as a categorical variable showed a statistically significant increase in cancer risk of 33% when the highest vs. the lowest quantile of the TyG index was considered (OR 1.33, 95% CI 1.22–1.45, *p* < 0.0001, n = 29) (Figure 3). Although substantial heterogeneity was observed among the studies (I^2^ = 77%, *p* < 0.0001), a leave-one-out sensitivity analysis showed that the pooled odds ratio remained stable—ranging from 1.31 (95% CI 1.21–1.43) when excluding Panigoro et al. 2021 [28] to 1.39 (95% CI 1.25–1.53) when excluding Son et al. 2024 [45]—with all estimates retaining statistical significance (Supplementary Figure S2). Stratifying the analysis by study design (case–control/cross-sectional vs. cohort) significantly reduced heterogeneity only in the case–control/cross-sectional subgroup (Table 2). In both subgroups, individuals in the highest TyG index category exhibited a significantly increased risk of cancer. Specifically, across nine cohort studies, participants in the highest TyG category had a 19% greater cancer risk compared with those in the lowest category (OR 1.19, 95% CI 1.10–1.29, *p* < 0.0001; n = 18) (Table 2). Further stratification showed that the data did not change significantly when only the prospective cohort studies were considered, while the retrospective studies showed a statistically non-significant increase in cancer risk (Table 2). Further subgroup analysis by tumor site revealed statistically significantly elevated risks for gastrointestinal (overall), colorectal (including and excluding adenomas), gastric, breast, and gynecological cancers in the highest TyG index group (Table 2). In the case of gastrointestinal and breast cancers, it was possible to further stratify the analysis by study design. The results indicate that a significant increase in risk for gastrointestinal cancer was evident in both case–control/cross-sectional studies and cohort studies (Table 2). On the other hand, for breast cancers, a significant increase in risk was highlighted in case–control/cross-sectional studies but not in cohort studies (Table 2). Finally, stratification by region showed a higher cancer risk in the Asian population (+59%) compared to the Western population (+22%) (Table 2).

**Figure 4:**
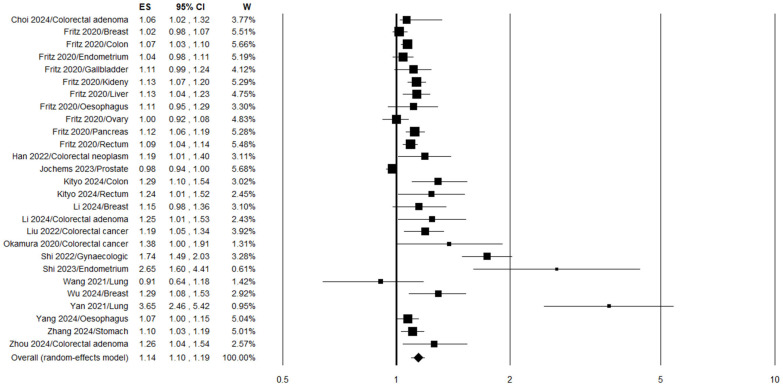
Forest plot for the meta-analysis regarding the association between the TyG index, analyzed as a continuous variable, and cancer risk [26,27,29,30,31,35,36,39,40,41,42,44,47,48,49,50,51].

Substantially similar results were obtained when the TyG index was evaluated as a continuous variable. In this case, the extent of the increase in cancer risk was more modest and equal to 14% in association with a one-unit increase in TyG (OR 1.14, 95% CI 1.10–1.19, *p* < 0.0001, n = 27) (Figure 4), with high heterogeneity (I2 = 84%, *p* < 0.0001) (Table 3). Sensitivity analysis supported the consistency of these findings, with the estimated cancer risk varying from OR 1.12 (95% CI 1.08–1.16) after excluding Shi et al. 2022 [36] to OR 1.15 (95% CI 1.10–1.20) after excluding other datasets, such as Wang et al. 2022 [29], with all estimates remaining statistically significant (Supplementary Figure S3). Data analysis stratified by study design (case–control/cross-sectional vs. cohort) revealed that a one-unit increase in TyG index was significantly associated with higher cancer risk in both subgroups (Table 3). Specifically, pooling data from nine cohort studies yielded a 9% increase in cancer risk (OR 1.09, 95% CI 1.05–1.12, *p* < 0.0001; n = 19) (Table 3). No reduction in heterogeneity was evident after stratifying the analysis according to study design, particularly in the case–control/cross-sectional group (I2 = 90%, *p* < 0.0001) (Table 3). Subgroup analysis by tumor site demonstrated a significant elevation in risk for gastrointestinal cancers (overall), colorectal cancers (including and excluding adenomas), esophageal cancers, and gynecological cancers (Table 3). Further stratification on the basis of study design demonstrated a significant increase in gastrointestinal cancer risk in both case–control/cross-sectional studies and cohort studies (Table 3). Again, stratification by region showed a higher cancer risk in the Asian population (+29%) compared to the Western population (+9%) (Table 3).

### 3.5. Publication Bias

Regarding the WMD analysis, no significant publication bias was detected by Egger’s and Begg’s tests (Egger’s test *p* = 0.060 and Begg’s test *p* = 0.484) or the funnel plot (Supplementary Figure S4).

Instead, clear publication bias was detected through Egger’s and Begg’s tests and visual inspection of funnel plot asymmetry when the TyG index was analyzed as either a categorical variable (Table 2 and Supplementary Figure S5) or as a continuous variable (Table 3 and Supplementary Figure S6). These findings persisted in many cases even after stratifying by study design and tumor site (Table 2 and Table 3). Applying the “trim and fill” method to adjust for publication bias, as proposed by Duval and Tweedie [52], the overall effect size estimates were 1.20 (95% CI 1.09–1.31, *p* < 0.0001, 11 studies imputed) when the TyG index was analyzed as a categorical variable and 1.08 (95% CI 1.03–1.13, *p* < 0.001, nine studies imputed) when analyzed as a continuous variable. Finally, when the TyG index was analyzed as a categorical variable, the fail-safe N was calculated at 996—well exceeding Rosenthal’s recommended threshold of (5k + 10 = 150) for our k = 28 estimates [53]—indicating that near a thousand null-effect studies would be needed to render the overall effect non-significant. Similarly, when the TyG index was analyzed as a continuous variable, the fail-safe N was calculated at 1095—well exceeding Rosenthal’s recommended threshold of (5k + 10 = 145) for our k = 27 estimates [53]. Thus, although large publication bias was observed, our data retain a certain degree of robustness.

## 4. Discussion

The TyG index is a well-validated surrogate marker of insulin resistance (IR). It demonstrated a strong correlation with the hyperinsulinemic–euglycemic clamp method and has been recognized for its predictive value in several metabolic disorders, including type 2 diabetes and metabolic syndrome [17]. Furthermore, the TyG index has been shown to be associated with various chronic degenerative diseases, such as cardiovascular disease and metabolic dysfunction-associated steatotic liver disease [17,54]. In the context of cancer risk, the TyG index presents distinct characteristics compared to traditional insulin resistance (IR) markers like HOMA-IR and the Matsuda Index. HOMA-IR is a well-established tool that primarily reflects fasting hepatic IR, while the Matsuda Index is considered a superior measure of whole-body insulin sensitivity, as it accounts for both hepatic and muscular components during a glucose challenge. However, the TyG index provides a unique metabolic perspective by incorporating triglyceride levels. This allows the index to capture “lipotoxicity,” a condition characterized by high levels of circulating free fatty acids that promote chronic low-grade inflammation and oxidative stress—factors that significantly contribute to DNA damage and tumor initiation [12]. From a clinical standpoint, the TyG index holds a significant advantage: it relies on standardized, low-cost biochemical parameters (glucose and triglycerides). In contrast, HOMA-IR and Matsuda Index require insulin assays, which lack international standardization and exhibit significant variability between different laboratory platforms. This makes the TyG index a more robust and accessible tool for large-scale epidemiological studies and for clinical settings where dynamic tests like the OGTT (required for the Matsuda Index) are not feasible. In this systematic review and meta-analysis, we provide robust evidence that elevated TyG index levels are significantly associated with an increased cancer risk. This effect was observed when the TyG index was evaluated both as a categorical variable and as a continuous variable. Furthermore, the increased cancer risk associated with the TyG index persisted after stratifying the analysis by study design. Despite notable heterogeneity across studies, the findings remained consistent through sensitivity and subgroup analyses, reinforcing their reliability. Interestingly, the observed associations between elevated TyG index and cancer risk were more pronounced in case–control and cross-sectional studies than in cohort studies. This could reflect biases related to reverse causality or selection in non-prospective designs. However, the association remained statistically significant in cohort studies as well (OR = 1.19 for categorical TyG; OR = 1.09 for continuous TyG), supporting the hypothesis that insulin resistance may precede cancer onset. This observation is particularly important and suggests that TyG may have a causal role in tumor development, acting as a risk factor for cancer. Our findings agree with the growing literature suggesting that metabolic dysregulation, particularly insulin resistance, contributes significantly to cancer development [55]. Notably, cancer patients exhibited significantly higher mean TyG levels than healthy controls (WMD = 0.23, *p* < 0.0001), indicating that participants with cancer had, on average, a 0.23 higher TyG index compared to those without cancer, suggesting that such metabolic imbalances may precede or accompany oncogenesis. Our results are in accordance with those reported in a recent study focused on the diagnostic performance of the TyG index in predicting cancer occurrence using ROC curve analysis, in which it was shown that cancer patients had a significantly higher TyG index than healthy subjects without cancer (mean difference: 0.34, 95% CI: 0.23–0.45) [56].

From a pathophysiological perspective, insulin resistance can promote a pro-oncogenic environment through multiple mechanisms. Compensatory hyperinsulinemia enhances cell proliferation and inhibits apoptosis by activating the insulin and insulin-like growth factor-1 (IGF-1) signaling pathways. These pathways stimulate the PI3K/Akt/mTOR axis, a key regulator of tumorigenesis and cancer cell survival [57]. Additionally, IR may contribute to chronic low-grade inflammation and oxidative stress, which are known promoters of DNA damage and tumor initiation [58].

Subgroup analyses by tumor site consistently revealed a significant association between elevated TyG index and increased risk of gastrointestinal cancers (including colorectal and gastric), as well as breast and gynecological malignancies. For gastrointestinal cancers, this relationship was evident in both case–control/cross-sectional and cohort studies. For breast cancer, however, the significant association was confined to non-cohort studies when the TyG index was considered as a categorical variable. While this manuscript was being written, two new meta-analyses were published regarding the association between TYG and breast and colon cancers, respectively [59,60]. A comparison of our data with that recently published showed complete concordance in the data for breast cancer [59]. In the case of colon cancer, however, small differences were found. Notably, the published meta-analysis focused only on cohort studies considering the incidence of colon cancer. Furthermore, the study also considered mortality from colon cancer and provided details regarding the dose-dependent relationship [60]. In any case, the paper suggested an increase of 29% in colorectal cancer risk associated with the fourth quartile when the TyG was analyzed as a categorical variable (using data from three studies) and an increase of 23% when the TyG was analyzed as a continuous variable (using data from four studies) [60]. In addition to its general predictive value, our findings suggest that the TyG index may exhibit varying degrees of organ specificity compared to traditional IR markers. The observed strong association between the TyG index and colorectal cancers confirms previous data indicating that higher HOMA-IR levels were significantly associated with an increased risk of colorectal adenoma [8]. In contrast, higher fasting insulin levels were not associated with breast cancer, although HOMA-IR levels were significantly higher in women with breast cancer compared to controls [7]. Our findings on gastrointestinal cancer, as well as breast cancer, are consistent with the hypothesis that these neoplasms may be highly sensitive not only to hyperinsulinemia but also to lipid-mediated inflammatory pathways. In the case of colon, elevated triglycerides—a key component of the TyG index—are closely linked to altered bile acid secretion and gut microbiota dysbiosis, which promote local carcinogenesis. In contrast, for prostate cancer and particularly endometrial cancer, our analysis shows less consistent results, while a significantly higher risk of EC was observed in women with elevated fasting insulin and HOMA-IR [9]. This observation suggests that these tumors might be driven by distinct hormonal pathways in which the insulin–lipid axis plays a secondary role. Overall, we may suggest that the predominance of adipose tissue in organs like the breast may provide a mechanistic link between elevated TyG index levels and tumor progression, given the role of adipocytes in insulin resistance and chronic inflammation.

An additional interesting finding of our study concerns ethnic disparities in the association between TyG and cancer risk. Specifically, the cancer risk estimate for Asian cohorts was significantly higher than that observed in European and US cohorts. These differences may be due to several factors, such as visceral adiposity, as Asian individuals tend to accumulate higher levels of visceral and ectopic fat compared to Caucasians, or the biological response to hyperinsulinemia may differ between ethnicities. Furthermore, differences in genetic polymorphisms related to lipid metabolism and lifestyle factors, including dietary habits, may somewhat exacerbate the impact of insulin resistance in Asian countries. Regardless, these observations highlight the need for ethnicity-specific cutoffs when using metabolic indices for cancer risk stratification in clinical practice.

Although publication bias was detected—confirmed through Egger’s and Begg’s tests and visual funnel plot asymmetry—the findings remained statistically robust after adjustment using the Duval and Tweedie trim-and-fill method [52]. Furthermore, the fail-safe N values (996 for categorical and 1095 for continuous analyses) far exceeded Rosenthal’s thresholds [53], indicating that a large number of hypothetical null studies would be needed to negate our results.

Several limitations of the present meta-analysis should be acknowledged. First, the observational nature of the included studies limits causal inference and leaves room for unmeasured confounding. Second, the heterogeneity in study design, population characteristics, and TyG measurement methods may have affected pooled estimates. Third, while some studies provided pre-diagnostic TyG levels, many did not, making it challenging to determine the temporal relationship. In addition, it must be recognized that TyG is certainly correlated with BMI, and therefore, the two indices may overlap somewhat; however, TyG certainly provides additional information on metabolic health. Nonetheless, the overall consistency of the associations, coupled with biological plausibility, supports the potential use of the TyG index as a simple, inexpensive marker for cancer risk stratification—particularly in populations with insulin resistance or metabolic syndrome. In addition, the exclusion of insulin from the TyG index calculation is one of its primary advantages for clinical and epidemiological use. Unlike glucose and triglycerides, which are subject to strict international standardization, insulin assays lack a universal reference standard, leading to significant inter-laboratory variability that can affect the reproducibility of HOMA-IR and Matsuda Index results. Furthermore, by incorporating triglycerides, the TyG index accounts for the metabolic impact of lipid-induced insulin resistance, which is a key driver of the pro-carcinogenic inflammatory state. This makes the TyG index not only a more accessible and cost-effective tool but also a more robust marker for assessing cancer risk in large, diverse populations.

## 5. Conclusions

Although our results evidence an increase in cancer risk associated with higher TyG index values, the low number of studies of the effect on tumors in different sites resulted in some cases not being statistically significant. Additional epidemiological studies with a cohort design are necessary to confirm these associations.

## Figures and Tables

**Table 1:** Characteristics of the studies included in the meta-analysis on the association between TyG index and cancer risk.

First Author Year Location Reference	Study Design and Name Population, Cases/Controls Incident Cases Follow-Up Age (year)	Measurement Method of TyG and Values	Cancer Site	TyG: Parametrization	OR/RR/HR (95% CI)	P for Trend	Matched or Adjusted Variables	NOS
Fritz et al. 2020 Norway, Sweden, Austria [26]	Cohort Metabolic Syndrome and Cancer Project (Me-Can) 2.0 510,471 subjects Incident cases: 16,052 Follow-up: 17.2 years, Age: 43.1 ± 10.6	Ln [triglyceride (mg/dL) × glucose (mg/dL)/2] TyG mean ± SD: 8.60 ± 0.60 Overall	Esophagus Colon Rectum Liver Gallbladder Pancreas Breast (postmenopausal) Endometrium Ovary Kidney (renal cell)	Quintile 1: <8.1 Quintile 5: >9.1 Continuous Quintile 5 Continuous Quintile 5 Continuous Quintile 5 Continuous Quintile 5 Continuous Quintile 5 Continuous Quintile 5 Continuous Quintile 5 Continuous Quintile 5 Continuous Quintile 5 Continuous	1.00 (Ref.) 1.27 (0.77–2.07) 1.11 (0.95–1.29) 1.14 (1.03–1.27) 1.07(1.03–1.10) 1.24 (1.08–1.42) 1.09(1.04–1.14) 1.29 (0.96–1.72) 1.13(1.04–1.23) 1.38 (0.95–1.99) 1.11(0.99–1.24) 1.37 (1.13–1.65) 1.12 (1.06–1.19) 1.07 (0.95–1.20) 1.02 (0.98–1.07) 1.22 (1.01–1.47) 1.04(0.98–1.11) 1.00 (0.80–1.25) 1.00 (0.92–1.08) 1.36 (1.13–1.63) 1.13(1.07–1.20)	0.186 --- <0.001 --- 0.001 --- 0.193 --- 0.176 --- 0.001 --- 0.334 --- 0.089 --- 0.937 --- <0.001 ---	Age, sex, smoking status, fasting status, cohort, decade of birth, BMI ^1^	9
Okamura et al. 2020 Japan [27]	Cohort NAGALA ^2^ 27,805 subjects Incident cases: 116 Follow-up: 4.4 years Age: 51.1 ± 9.3 Incident cases 45.6 ± 10.1 Cohort	Ln [triglyceride (mg/dL) × glucose (mg/dL)/2] TyG mean ± SD: 8.2 ± 0.7 Cohort 8.4 ± 0.7 Cases	Colorectal	Continuous	1.38 (1.00–1.91)	0.049	Age, sex, BMI, smoking status, alcohol consumption, exercise, systolic blood pressure and serum creatinine	9
Panigoro et al. 2021 Indonesia [28]	Case–control (HB ^3^) Cases: 212 Controls: 212 Age: 48 (range: 22–78) Cases 46 (range: 22–75) Controls	Ln [triglyceride (mg/dL) × glucose (mg/dL)/2] TyG median (CI 95%): 8.30 (7.09–10.84) Controls 8.65 (7.3–10.9) Cases	Breast	Quartile 1 Quartile 4	1.00 (Ref.) 2.42 (1.77–3.31) 2.93 (1.72–4.98)	---	---	8
Wang et al. 2021 The United Kingdom [29]	Cohort UK Biobank 324,334 subjects Incident case: 1593 Follow-up: 9 years Age: 61.08 (mean) Incident cases 55.805 (mean) Cohort	Ln [triglyceride (mg/dL) × glucose (mg/dL)/2] TyG mean ± SD: 8.667 ± 0.541 Cohort 8.668 ± 0.529 Cases	Lung	<8.639 >8.639 Continuous	1.00 (Ref.) 0.966 (0.850–1.097) 0.911 (0.640–1.182)	0.589 0.499	Age, sex, region, Townsend deprivation score, smoking status, alcohol intake frequency, BMI, waist hip rate, hypertension, total cholesterol, LDL ^4^, HDL ^5^, HbA1c ^6^	9
Yan et al. 2021 China [30]	Case–control (HB) Cases: 791 Controls: 787 Age: 61.75 ± 10.68 Cases 59.93 ± 10.73 Controls	Ln [triglyceride (mg/dL) × glucose (mg/dL)/2] TyG mean ± SD: 8.00 ± 0.45 Controls 8.42 ± 0.55 Cases	Lung	Continuous	3.651 (2.461–5.417)	<0.001	Age, sex, smoking, BMI, hypertension, WBCC, Neutrophil count, TC, LDL-C, HDL-C, uric acid.	9
Han et al. 2022 China [31]	Cross-sectional Cases: 1462 Controls: 947 Age: 59.22 ± 10.36 Cases 54.04 ± 11.87 Controls	Ln [triglyceride (mg/dL) × glucose (mg/dL)/2] TyG mean ± SD: 8.63 ± 0.63 Controls 8.71 ± 0.60 Cases	Colorectal neoplasm	Quartile 1 Quartile 4 Continuous	1.00 (Ref.) 1.35 (1.02–1.77) 1.19 (1.01–1.40)	--- 0.038	Age, sex, family history, FOBT ^7^	8
Li et al. 2022 China [32]	Cohort Kailuan Study 93,659 subjects Incident case: 586 Follow-up: 13.02 years Age: 51.44 ± 12.45	Ln [triglyceride (mg/dL) × glucose (mg/dL)/2]	Colorectal	<8.59 ≥8.59 Continuous	1.00 (Ref.) 1.41 (1.17–1.67) 1.21 (1.06–1.37)	<0.001 0.006	Age, sex, family income, educational background, WC, TC, smoking, drinking, physical activity, sedentary lifestyle, tea consumption, high-fat diet, hypertension, diabetes, family history of cancer.	7
Kim et al. 2022 Korea [33]	Cohort 83,853 subjects Incident cases: 186 Follow-up: 14 years Age: 48.6 ± 11.4	Ln [triglyceride (mg/dL) × glucose (mg/dL)/2] TyG mean ± SD: 9.23 ± 0.59 Cohort 9.48 ± 0.63 Cases	Stomach	Quartile 1 Quartile 4	1.00 (Ref) 2.363 (1.391–4.014)	---	Age, male sex, obesity, smoking, hypertension, DM, and H. pylori infection	9
Alkurt et al. 2022 Turkey [34]	Cross-sectional Cases: 254 Controls: 128 Age: 51.55 ± 11.94 Controls 50.19 ± 13.24 Cases	[triglyceride (mg/dL) × glucose (mg/dL)/2] TyG mean ± SD: ^8^ 9.02 ± 8.68 Controls 9.27 ± 9.19 Cases	Thyroid	<8.74 ^7^ >8.74	1.00 (Ref) 2.147 (1.387–3.323)	---	Age, sex, operation times, presence of neck dissection, TSH ^9^, FT3 ^10^, FT4 ^11^, fasting blood glucose and triglyceride levels	8
Liu et al. 2022 China [35]	Cohort Kailuan study 93,659 subjects Incident case: 593 Follow up: 13.02 years Age: 51.44 ± 12.45	Ln [triglyceride (mg/dL) × glucose (mg/dL)/2] TyG mean ± SD: 8.66 ± 0.69 Overall	Colorectal	Quartile 1 Quartile 4 Continuous	1.00 (Ref) 1.50 (1.19–1.91) 1.19 (1.05–1.34)	0.004 0.008	Age, sex, family income, education, marital status, WC, TC, smoking, drinking, physical activity, sedentary lifestyle, tea consumption, salt intake, high-fat diet, hypertension, family history of cancer, diabetes	7
Shi et al. 2022 China on USA dataset [36]	Cross-sectional National Health and Nutrition Examination Survey (NHANES) Cases: 306 breast 152 cervix 45 ovarian 83 endometrium Controls: 10,880	Ln [triglyceride (mg/dL) × glucose (mg/dL)/2] TyG range: 6.19–11.96 Overall	Breast Cervix Ovarian Endometrium Combined	Quartile 1 Quartile 4 Quartile 4 Quartile 4 Quartile 4 Continuous	1.00 (Ref) 2.25 (1.50–3.37) 1.68 (0.99–2.84) 3.734 (1.01–13.87) 2.424 (1.14–5.16) 1.740 (1.492–2.029)	--- --- --- --- <0.001	Age, race, marital status, BMI, HDL, LDL, education, age at menarche, age at menopause, diabetes, hypertension, breastfeeding history	8
Li et al. 2023 China [37]	Case–control (HB) Cases: 136 Controls: 631 Age: 46 Controls (median) 71 Cases (median)	Ln [triglyceride (mg/dL) × glucose (mg/dL)/2] TyG mean (CI 95%): 8.92 (8.5–9.41) Cases 8.29 (7.94–8.70) Controls	Prostate	Quartile 1 Quartile 4	1.00 (Ref) 28.867 (9.499–87.727)	---	Age, education, drinking, alkaline phosphatase, low-density lipoprotein, blood calcium, blood potassium, total cholesterol	8
Zhou et al. 2024 China [38]	Cross-sectional Cases: 136 Controls: 180 Age: 65.10 ± 8.51 Controls 70.73 ± 9.80 Cases	Ln [triglyceride (mg/dL) × glucose (mg/dL)/2] TyG mean ± SD: 8.93 ± 0.69 Cases 8.74 ± 0.58 Controls	Prostate	Quartile 1 Quartile 4	1.00 (Ref) 2.854 (1.20–6.79)	---	Age, initial PSA, smoking history, alcohol consumption, family history of cancer, BMI, TC, and LDL	8
Jochems et al. 2023 Sweden [39]	Cohort (Pooled four cohorts: VIP, MONICA, MDCS, MPP) 56,897 subjects Incident case: 3325 Age: 51.44 ± 12.45	Ln [triglyceride (mg/dL) × glucose (mg/dL)/2] TyG mean ± SD: 8.55 ± 0.5 Overall	Prostate	Tertile1 Tertile3 Continuous	1.00 (Ref) 0.92 (0.84–1.00) 0.98 (0.94–1.00)	0.05 ---	Age, history of diabetes, country of birth, education, BMI, smoking status at baseline	9
Wu et al. 2024 China [40]	Cross-sectional REACTION study 141,375 Subjects Cases: 809 Age: 58.02 ± 8.74 Cases 56.36 ± 9.31 Cohort	Ln [triglyceride (mg/dL) × glucose (mg/dL)/2] TyG mean (CI 95%): 8.72 (8.39–9.14) Cases 8.63 (8.28–9.02) Cohort	Breast	Quartile 1 Quartile 4 Continuous	1.00 (Ref) 1.61 (1.19–2.17) 1.29 (1.08–1.53)	<0.0001 ---	Age, BMI, smoking, drinking, physical activity, family history of breast cancer, healthy diet, 2h-PG, HbA1c and HDL-C, age at menarche, menopausal status, number of childbirths, breastfeeding	9
Zhou et al. 2024 China [41]	Cohort 1538 subjects Incident case: 876 Age: 61.0 ± 5.46 Cases 59.9 ± 5.21 Controls	Ln [triglyceride (mg/dL) × glucose (mg/dL)/2] TyG mean ± SD: 8.67 ± 0.63 Cases 8.60 ± 0.57 Controls	Colorectal adenoma	Quartile 1 Quartile 4 Continuous	1.00 (Ref) 3.36 (1.44–7.73) 1.26 (1.04–1.54)	--- 0.019	Age, sex, overweight, smoking, drinking, diabetes, family history of CRC, aspirin use, FIT test results.	7
Shi et al. 2023 China [42]	Case–control (HB) Cases: 300 Controls: 300 Age: 58.6 ± 10.2 Cases 50.1 ± 14.0 Controls	Ln [triglyceride (mg/dL) × glucose (mg/dL)/2] TyG mean ± SD: 8.55 ± 0.57 Cases 7.97 ± 0.52 Controls	Endometrium	Continuous	2.65 (1.60–4.41)	<0.001	Age, abortion, age at first menarche, BMI, CRP, hypertension, HDL-C, LDL-C, menopausal status, neutrophil count, parturition, WBC	7
Zhang et al. 2024 China [43]	Cross-sectional Controls: 2111 Case: 477 Age: 51.40 ± 10.68 Cases 40.09 ± 11.00 Controls	Ln [triglyceride (mg/dL) × glucose (mg/dL)/2] TyG mean ± SD: 8.44 ± 0.55 Cases 8.24 ± 0.57 Controls	Breast	Quartile 1 Quartile 4	1.00 (Ref) 1.43 (1.01–2.02)	--	Age, BMI, smoking, drinking, hypertension, family history of malignancy, age at menarche, hormonal contraception	8
Li et al. 2024 China [44]	Cross-sectional Cases: 690 Controls: 2155 Age: 55.11 (9.65)	Ln [triglyceride (mg/dL) × glucose (mg/dL)/2] TyG mean ± SD: 8.62 ± 0.64 Cases 8.52 ± 0.67 Controls	Colorectal adenoma	Quartile 1 Quartile 4 Continuous	1.00 (Ref) 1.481 (1.022–2.146) 1.245 (1.013–1.529)	0.026 0.037	Age, sex, systolic blood pressure, history of cancer, hypertension, hyperglycemia, dyslipidemia, smoking, family history of colorectal cancer, high-density lipoprotein cholesterol	7
Son et al. 2024 Korea [45]	Cohort 314,141 subjects Incident case: 6112 Age: 58.8 Follow-up: 10 years	Ln [triglyceride (mg/dL) × glucose (mg/dL)/2] TyG mean ± SD: 8.68 ± 0.13 Overall	Colorectal	Quartile 1 Quartile 4	1.00 (Ref) 1.16 (1.07–1.25)	---	Age, sex, income, residence, hypertension, diabetes, dyslipidemia, Charlson comorbidity index, BMI, hemoglobin level, glomerular filtration rate, smoking, alcohol, exercise	9
Zha et al. 2024 China on USA dataset [46]	Cross-sectional NHANES Cases: 187 Controls: 21,411 Age: 73.0 years (Cases) 50.0 years (Controls)	Ln [triglyceride (mg/dL) × glucose (mg/dL)/2]	Stomach	Quartile 1 Quartile 4	1.00 (Ref) 2.082 (1.016–4.269)	---	Age, sex, education level, race, smoking, drinking	8
Yang et al. 2024 UK [47]	Cohort UK Biobank 388,900 subjects Incident cases: 779 Age: 57 (50–63) Follow-up: 13 years	Ln [triglyceride (mg/dL) × glucose (mg/dL)/2] TyG mean (CI 95%): 8.9 (8.5–9.2) Cases 8.7 (8.3–9.1) Cohort	Esophagus	Quartile 1 Quartile 4 Continuous	1.00 (Ref) 1.13 (0.91–1.40) 1.07 (1.00–1.15)	0.16	Age, sex, ethnicity, Townsend deprivation index, Metabolic Equivalent of Task (MET), smoking, alcohol, diabetes mellitus, hypertension, insulin, fasting time, diet score	9
Kityo et al. 2024 Korea [48]	Cohort 98,800 subjects Incident case: 699 Age: 40–69 years Follow-up: 10.6 years	Ln [triglyceride (mg/dL) × glucose (mg/dL)/2] TyG mean: 8.48 Overall	Colorectal Colon Rectum	Continuous Continuous Continuous	1.28 (1.12–1.46) 1.29 (1.10–1.54) 1.24 (1.01–1.52)	--- --- ---	Age, sex, educational level, monthly income, smoking, drinking, regular physical exercise, BMI, fruit and vegetable intake, total red meat intake	9
Choi et al. 2024 Korea [49]	Cross-sectional Cases: 920 Controls: 3547 Age: 38.41 ± 6.36 Controls 41.36 ± 5.58 Cases	Ln [triglyceride (mg/dL) × glucose (mg/dL)/2] TyG mean ± SD: 8.51 ± 0.71 Cases 8.32 ± 0.61 Controls	Colorectal adenoma	Continuous	1.064 (1.023–1.325)	0.021	Age, sex, BMI, fasting glucose, HDL-C, LDL-C, TG, alcohol, Smoke, hypertension, diabetes, dyslipidemia	8
Zhang et al. 2024 China [50]	Case–control (PB ^12^) Cases: 215 Controls: 827 Age: 66 (60–72) Cases 65 (61–71) Controls	Ln [triglyceride (mg/dL) × glucose (mg/dL)/2]	Stomach	Continuous	1.104 (1.028–1.186)	---	Sex, BMI, hypertension.	8
Li et al. 2024 China [51]	Cohort Kailuan Study 27,604 subjects Incident cases: 375 Age: 47.53 ± 11.95 years Follow-up: 12.90 ± 2.03 years	Ln [triglyceride (mg/dL) × glucose (mg/dL)/2]	Breast	Tertile1 Tertille 3 Continuous	1.00 (Ref) 1.21 (0.91–1.60) 1.15 (0.98–1.36)	0.21 0.09	Age, systolic blood pressure, waist-hip ratio, triglycerides, total cholesterol, frequency of physical exercise, smoking, alcohol consumption, salt intake	7

^1^ Body Mass Index; ^2^ NAfld in the Gifu Area Longitudinal Analysis; ^3^ hospital-based; ^4^ low-density lipoprotein cholesterol; ^5^ high-density lipoprotein cholesterol; ^6^ glycated hemoglobin; ^7^ Fecal Occult Blood Test; ^8^ Ln of TyG has been calculated; ^9^ serum thyroid-stimulating hormone; ^10^ free triiodothyronine; ^11^ free thyroxine; ^12^ population-based.

**Table 2:** Results of stratified analysis of cancer risk estimates associated with the TyG index, calculated as a categorical variable.

	N° of Studies	References	N° of Estimates	Combined Risk Estimate		Test of Heterogeneity			Publication Bias	
				Value (95% CI)	*p*	Q	I^2^%	*p*	*p* (Egger)	*p* (Begg)
Overall	17	[26,28,29,31,33,35,36,38,39,40,41,43,44,45,46,47,51]	29	1.33 (1.22–1.45)	<0.0001	120.63	76.79	<0.0001	<0.001	<0.001
Study design										
Case–control/cross-sectional	8	[28,31,36,38,40,43,44,46]	11	1.78 (1.51–2.09)	<0.0001	14.84	32.62	0.138	0.090	0.052
Cohort	9	[26,29,33,35,39,41,45,47,51]	18	1.19 (1.10–1.29)	<0.0001	61.58	72.36	<0.0001	0.007	0.037
Prospective	7	[26,29,35,39,41,47,51]	16	1.18 (1.08–1.29)	<0.0001	52.68	71.53	<0.0001	0.004	0.072
Retrospective	2	[33,45]	2	1.57 (0.79–3.14)	0.197	6.78	85.25	0.009	---	---
Tumor site										
Gastrointestinal cancers	9	[26,31,33,35,41,44,45,46,47]	13	1.29 (1.18–1.41)	<0.0001	23.57	49.08	0.023	0.0001	0.010
Case–control/cross-sectional	3	[31,44,46]	3	1.44 (1.17–1.78)	0.001	1.25	0.00	0.536	0.002	0.117
Cohort	6	[26,33,35,41,45,47]	10	1.27 (1.16–1.39)	<0.0001	19.73	54.39	0.020	0.006	0.016
Prospective	4	[26,35,41,47]	8	1.27 (1.15–1.40)	<0.0001	12.19	42.58	0.094	0.046	0.083
Retrospective	2	[33,45]	2	1.57 (0.79–3.14)	0.197	6.78	85.25	0.009	---	---
Colorectal (with adenoma)	6	[26,31,35,41,44,45]	7	1.26 (1.14–1.39)	<0.0001	13.00	53.83	0.043	0.002	0.011
Colorectal (without adenoma)	3	[26,35,45]	4	1.20 (1.11–1.30)	<0.0001	0.05	40.57	0.168	0.089	0.174
Esophagus	2	[26,47]	2	1.15 (0.94–1.40)	0.162	0.18	0.00	0.671	----	----
Stomach	2	[33,46]	2	2.26 (1.48–3.46)	0.0002	0.08	0.00	0.781	----	----
Breast	6	[26,28,36,40,43,51]	6	1.56 (1.17–2.08)	0.003	35.19	85.79	<0.0001	0.036	0.091
Case–control/cross-sectional	4	[28,36,40,43]	4	1.87 (1.45–2.41)	<0.0001	6.66	54.98	0.083	0.821	1.000
Cohort	2	[26,51]	2	1.09 (0.98–1.21)	0.120	0.62	0.00	0.430	----	----
Prostate	2	[38,39]	2	1.49 (0.50–4.45)	0.478	6.49	84.59	<0.0001	----	----
Gynecological cancers	2	[26,36]	5	1.36 (1.02–1.82)	0.034	10.38	61.45	0.035	0.063	0.142
Endometrium	2	[26,36]	2	1.55 (0.82–2.96)	0.179	2.99	66.58	0.084	----	----
Ovary	2	[26,36]	2	1.64 (0.47–5.72)	0.439	3.78	73.52	0.052	----	----
Region										
Asia	11	[28,31,33,35,38,40,41,43, 44,45,51]	11	1.59 (1.33–1.90)	<0.0001	40.74	75.46	<0.0001	0.001	0.052
Europe/USA	6	[26,29,36,39,46,47]	18	1.22 (1.11–1.34)	<0.0001	63.89	73.39	<0.0001	<0.001	0.021

**Table 3:** Results of stratified analysis of cancer risk estimates associated with the TyG index, calculated as a continuous variable.

	N° of Studies	References	N° of Estimates	Combined Risk Estimate		Test of Heterogeneity			Publication Bias	
				Value (95% CI)	*p*	Q	I^2^%	*p*	*p* (Egger)	*p* (Begg)
Overall	17	[26,27,29,30,31,35,36,39,40,41,42,44,47,48,49,50,51]	27	1.14 (1.10–1.19)	<0.0001	161.10	83.86	<0.0001	<0.001	0.005
Study design										
Case–control/cross-sectional	8	[30,31,36,40,42,44,49,50]	8	1.46 (1.21–1.76)	<0.0001	71.46	90.20	<0.0001	0.026	0.048
Cohort	9	[26,27,29,35,39,41,47,48,51]	19	1.09 (1.05–1.12)	<0.0001	61.53	70.75	<0.0001	0.010	0.172
Tumor site										
Gastrointestinal cancers	10	[26,27,31,35,41,44,47,48,49,50]	15	1.11 (1.08–1.14)	<0.0001	16.88	17.07	0.263	<0.001	0.002
Case–control/cross-sectional	4	[31,44,49,50]	4	1.12 (1.06–1.18)	0.0001	2.29	0.00	0.515	0.339	0.174
Cohort	6	[26,27,35,41,47,48]	11	1.11 (1.08–1.14)	<0.0001	14.27	29.92	0.161	<0.001	0.010
Colorectal (with adenoma)	8	[26,27,31,35,41,44,48,49]	10	1.13 (1.08–1.19)	<0.0001	15.40	41.54	0.081	<0.001	0.060
Colorectal (without adenoma)	4	[26,27,35,48]	6	1.13 (1.07–1.19)	<0.0001	10.63	52.97	0.059	0.001	0.188
Esophagus	2	[26,48]	2	1.08 (1.01–1.15)	0.023	0.18	0.00	0.669	----	----
Breast	3	[26,40,51]	3	1.13 (0.97–1.30)	0.111	8.07	75.22	0.018	0.187	0.117
Gynecological cancers	3	[26,36,42]	4	1.34 (1.05–1.71)	0.020	53.94	94.44	<0.0001	0.168	0.497
Endometrium	2	[26,42]	2	1.60 (0.64–4.00)	0.312	12.88	92.24	0.0003	----	----
Lung	2	[29,30]	2	1.81 (0.47–7.07)	0.391	29.65	96.63	<0.0001	----	----
Region										
Asia	12	[27,30,31,35,40,41,42,44,48, 49,50,51]	13	1.29 (1.17–1.42)	<0.0001	51.03	76.49	<0.0001	0.001	0.005
Europe/USA	5	[26,29,36,38,47]	14	1.09 (1.04–1.13)	<0.0001	83.79	84.49	<0.0001	0.080	0.477

## Data Availability

Data are contained within the article. Additional information is available from the corresponding author upon reasonable request.

## Supplementary Materials

The following supporting information can be downloaded at https://www.mdpi.com/article/10.3390/jpm16050274/s1: Figure S1: Sensitivity analysis for the WMD obtained comparing mean TyG index levels between cancer patients and healthy controls. Figure S2: Sensitivity analysis on the association between TyG index, analyzed as a categorical variable, and cancer risk. Figure S3: Sensitivity analysis on the association between the TyG index, analyzed as a continuous variable, and cancer risk. Figure S4: Funnel plot on the WMD obtained comparing mean TyG index levels between cancer patients and healthy controls. Figure S5: Funnel plot on the association between the TyG index, analyzed as a categorical variable, and cancer risk. Figure S6: Funnel plot on the association between the TyG index, analyzed as a continuous variable, and cancer risk. Table S1: Methodological quality of case–control/cross-sectional studies included in the meta-analysis. Table S2: Methodological quality of cohort studies included in the meta-analysis, File S1: the PRISMA checklist.

## Abbreviations

The following abbreviations are used in this manuscript:

TyG	Triglyceride–Glucose Index
T2DM	Type 2 Diabetes Mellitus
PICOS	Population, Intervention, Comparison, Outcomes, Study design
NOS	Newcastle–Ottawa Scale
RR	Relative Risk
OR	Odds Ratio
HR	Hazard Risk
WMD	Weighted Mean Difference
