# Supplementary material for: Association Between Triglyceride–Glucose Index and Risk of Cancer: A Systematic Review and Meta-Analysis
Source: J Pers Med. 2026 May 20;16(5):274. doi: 10.3390/jpm16050274 (PMC13208625; doi:10.3390/jpm16050274)
Supplement: Supplementary file 1 [file jpm-16-00274-s001.zip › jpm-4262264-supplementary.pdf]

Supplementary Materials

Table S1. Methodological quality of case-control/cross-sectional studies included in the meta-analysis *.

First Author Year [Reference]	Adequate Definition of Cases	Representativeness of Cases	Selection of Control Subjects	Definition of Control Subjects	Control for Important Factor or Additional Factor †	Exposure Assessment	Same Method of Ascertainment for All Subjects	Non Response Rate ‡	Total Quality Scores (0-9)
Panigoro 2021 [28]	*	*		*	**	*	*	*	8
Yan 2021[30]	*	*	*	*	**	*	*	*	9
Han 2022 [31]	*	*		*	**	*	*	*	8
Alkurt 2022 [34]	*	*		*	**	*	*	*	8
Shi 2022 [36]	*	*	*		**	*	*	*	8
Li 2023 [37]	*	*	*	*	*	*	*	*	8
Zhou 2024 [38]	*	*		*	**	*	*	*	8
Wu 2024 [40]	*	*	*	*	**	*	*	*	9
Shi 2023 [42]	*	*	*		*	*	*	*	7
Zhang 2024 [43]	*	*		*	**	*	*	*	8
Li 2024 [44]	*	*			**	*	*	*	7
Zha 2024 [46]	*	*	*		**	*	*	*	8
Choi 2024 [49]	*	*	*	*	*	*	*	*	8
Zhang 2024 [50]	*	*	*		**	*	*	*	8

*A study could be awarded a maximum of one star for each item except for the item Control for important factor or additional factor. † A maximum of 2 stars could be awarded for this item. Studies that controlled for age received one star, whereas studies that controlled for other important confounders (smoking and total energy intake/BMI) received an additional star. ‡ One star was assigned if there was no significant difference in the response rate between control subjects and cases by using the chi-square test ($p < 0.05$).

Table S2. Methodological quality of cohort studies included in the meta-analysis *.

First Author Year [Reference]	Representativeness of the Exposed Cohort	Selection of the Unexposed Cohort	Ascertainment of Exposure	Outcome of Interest Not Present at Start of Study	Control for Important Factor or Additional Factor †	Assessment of Outcome	Follow-Up Long Enough for Outcomes to Occur ‡	Adequacy of Follow-Up of Cohorts §	Total Quality Scores (0-9)
Fritz 2020 [26]	*	*	*	*	**	*	*	*	9
Okamura 2020 [27]	*	*	*	*	**	*	*	*	9
Wang 2021 [29]	*	*	*	*	**	*	*	*	9
Li 2022 [32]		*	*	*	**	*	*		7
Kim 2022 [33]	*	*	*	*	**	*	*	*	9
Liu 2022 [35]		*	*	*	**	*	*		7
Jochems 2023 [39]	*	*	*	*	**	*	*	*	9
Zhu 2024 [41]	*	*	*	*	**	*			7
Son 2024 [45]	*	*	*	*	**	*	*	*	9
Yang 2024 [47]	*	*	*	*	**	*	*	*	9
Kityo 2024 [48]	*	*	*	*	**	*	*	*	9
Li 2024 [51]		*	*	*	**	*	*		7

*A study could be awarded a maximum of one star for each item except for the item Control for important factor or additional factor. † A maximum of 2 stars could be awarded for this item. Studies that controlled for age received one star, whereas studies that controlled for other important confounders (smoking and total energy intake/BMI) received an additional star. ‡ A cohort study with a follow-up time > 6 years was assigned one star. § A cohort study with a follow-up rate > 75% was assigned one star.

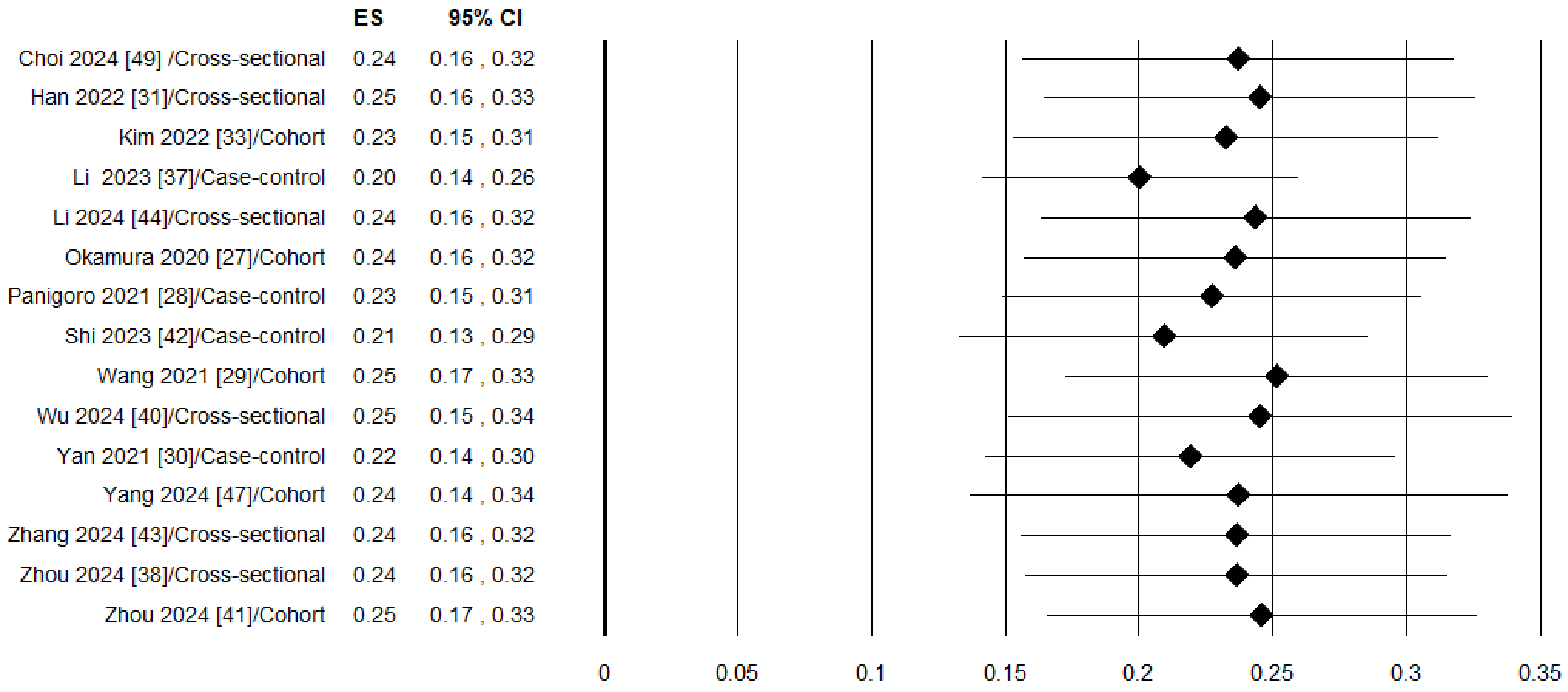
ES
95% CI
Choi 2024 [49] /Cross-sectional 0.24 0.16 , 0.32
Han 2022 [31]/Cross-sectional 0.25 0.16 , 0.33
Kim 2022 [33]/Cohort 0.23 0.15 , 0.31
Li 2023 [37]/Case-control 0.20 0.14 , 0.26
Li 2024 [44]/Cross-sectional 0.24 0.16 , 0.32
Okamura 2020 [27]/Cohort 0.24 0.16 , 0.32
Panigoro 2021 [28]/Case-control 0.23 0.15 , 0.31
Shi 2023 [42]/Case-control 0.21 0.13 , 0.29
Wang 2021 [29]/Cohort 0.25 0.17 , 0.33
Wu 2024 [40]/Cross-sectional 0.25 0.15 , 0.34
Yan 2021 [30]/Case-control 0.22 0.14 , 0.30
Yang 2024 [47]/Cohort 0.24 0.14 , 0.34
Zhang 2024 [43]/Cross-sectional 0.24 0.16 , 0.32
Zhou 2024 [38]/Cross-sectional 0.24 0.16 , 0.32
Zhou 2024 [41]/Cohort 0.25 0.17 , 0.33
0
0.05
0.1
0.15
0.2
0.25
0.3
0.35

Figure S1

	ES	95% CI
Fritz 2020 [26] /Breast	1.38	1.26 , 1.52
Fritz 2020 [26] /Colon	1.38	1.25 , 1.52
Fritz 2020 [26] /Endometrium	1.37	1.25 , 1.50
Fritz 2020 [26] /Gallbladder	1.36	1.24 , 1.49
Fritz 2020 [26] /Kideny	1.36	1.24 , 1.49
Fritz 2020 [26] /Liver	1.36	1.24 , 1.49
Fritz 2020 [26] /Oesophagus	1.36	1.24 , 1.49
Fritz 2020 [26] /Ovary	1.38	1.26 , 1.51
Fritz 2020 [26] /Pancreas	1.36	1.24 , 1.49
Fritz 2020 [26] /Rectum	1.37	1.25 , 1.51
Han 2022 [31] /Colorectal neoplasm	1.36	1.24 , 1.49
Jochems 2023 [39] /Prostate	1.37	1.26 , 1.49
Kim 2022 [33] /Stomach	1.34	1.23 , 1.46
Li 2024 [44]/Colorectal adenoma	1.35	1.24 , 1.48
Li 2024 [51]/Breast	1.37	1.25 , 1.50
Liu 2022 [35]/Colorectal cancer	1.35	1.23 , 1.48
Panigoro 2021 [28]/Breast	1.31	1.21 , 1.43
Shi 2022 [36]/Breast	1.33	1.22 , 1.45
Shi 2022 [36]/Cervix	1.35	1.23 , 1.48
Shi 2022 [36]/Endometrium	1.35	1.23 , 1.47
Shi 2022 [36]/Ovary	1.35	1.24 , 1.48
Son 2024 [45]/Colorectal cancer	1.39	1.25 , 1.53
Wu 2024 [40]/Breast	1.35	1.23 , 1.47
Yang 2024 [47]/Oesophagus	1.37	1.25 , 1.51
Zha 2024 [46]/Stomach	1.35	1.23 , 1.48
Zhang 2024 [43] /Breast	1.36	1.24 , 1.49
Zhou 2024 [38] /Prostate	1.35	1.23 , 1.47
Zhou 2024 [41] /Colorectal adenoma	1.34	1.23 , 1.47

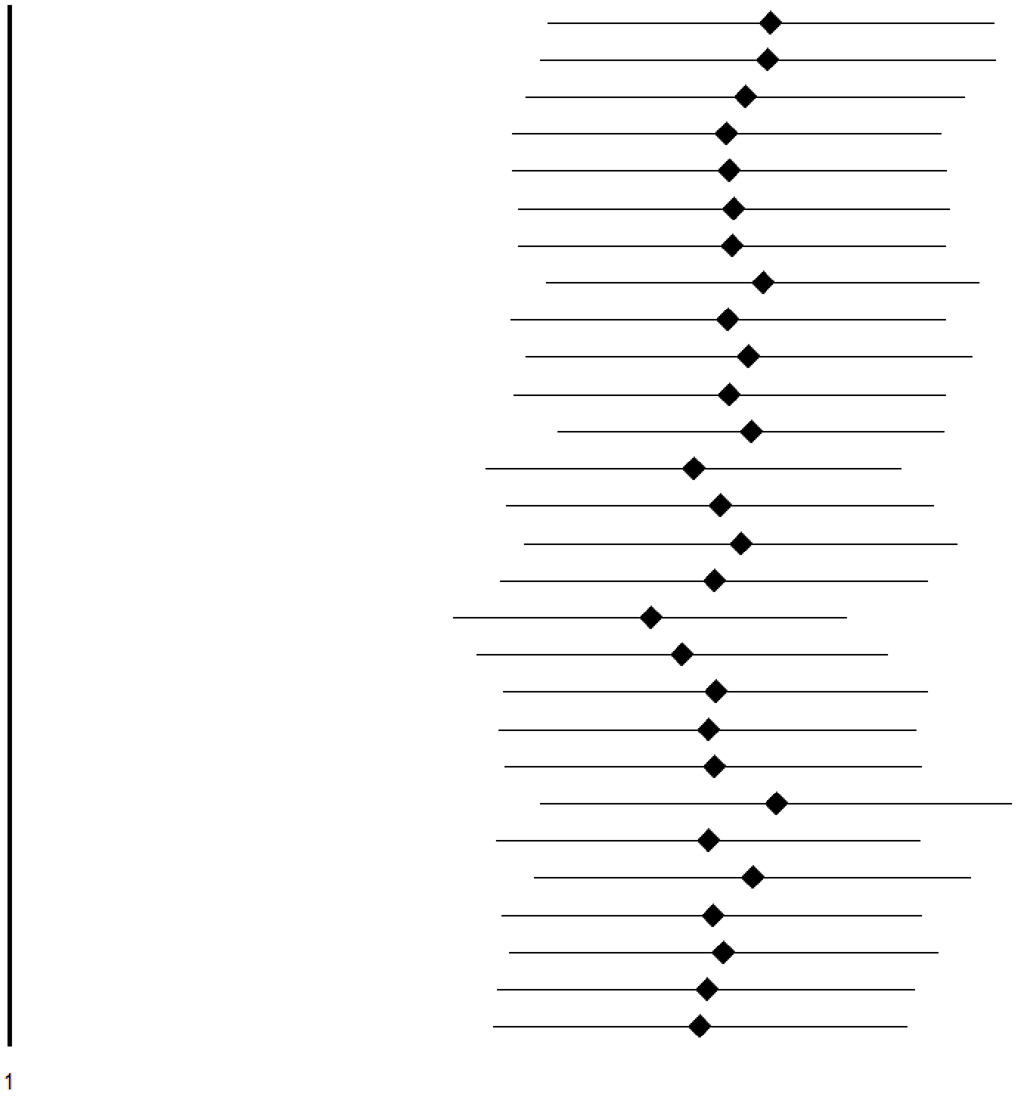

Figure S2

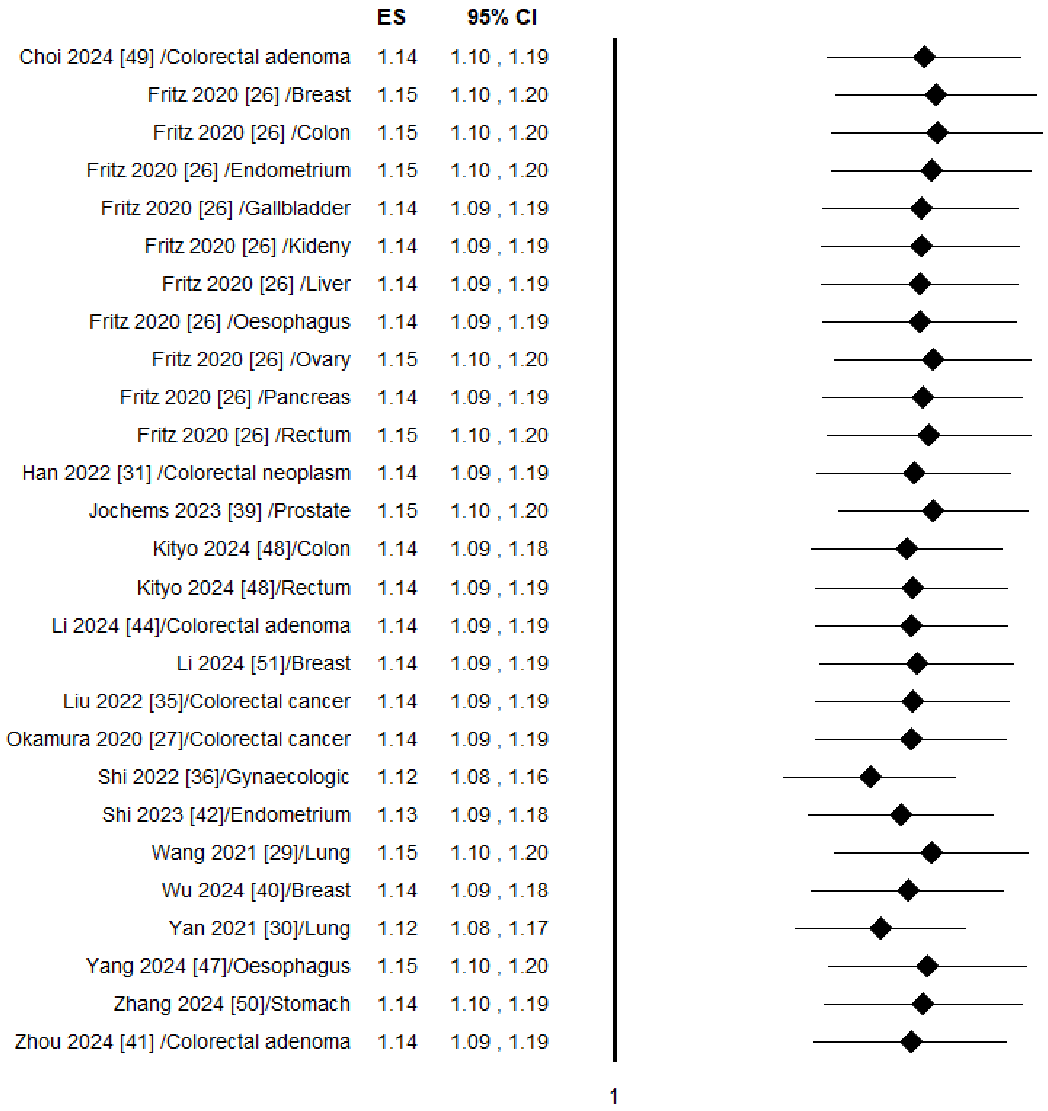
ES
95% CI
Choi 2024 [49] /Colorectal adenoma 1.14 1.10 , 1.19
Fritz 2020 [26] /Breast 1.15 1.10 , 1.20
Fritz 2020 [26] /Colon 1.15 1.10 , 1.20
Fritz 2020 [26] /Endometrium 1.15 1.10 , 1.20
Fritz 2020 [26] /Gallbladder 1.14 1.09 , 1.19
Fritz 2020 [26] /Kideny 1.14 1.09 , 1.19
Fritz 2020 [26] /Liver 1.14 1.09 , 1.19
Fritz 2020 [26] /Oesophagus 1.14 1.09 , 1.19
Fritz 2020 [26] /Ovary 1.15 1.10 , 1.20
Fritz 2020 [26] /Pancreas 1.14 1.09 , 1.19
Fritz 2020 [26] /Rectum 1.15 1.10 , 1.20
Han 2022 [31] /Colorectal neoplasm 1.14 1.09 , 1.19
Jochems 2023 [39] /Prostate 1.15 1.10 , 1.20
Kityo 2024 [48]/Colon 1.14 1.09 , 1.18
Kityo 2024 [48]/Rectum 1.14 1.09 , 1.19
Li 2024 [44]/Colorectal adenoma 1.14 1.09 , 1.19
Li 2024 [51]/Breast 1.14 1.09 , 1.19
Liu 2022 [35]/Colorectal cancer 1.14 1.09 , 1.19
Okamura 2020 [27]/Colorectal cancer 1.14 1.09 , 1.19
Shi 2022 [36]/Gynaecologic 1.12 1.08 , 1.16
Shi 2023 [42]/Endometrium 1.13 1.09 , 1.18
Wang 2021 [29]/Lung 1.15 1.10 , 1.20
Wu 2024 [40]/Breast 1.14 1.09 , 1.18
Yan 2021 [30]/Lung 1.12 1.08 , 1.17
Yang 2024 [47]/Oesophagus 1.15 1.10 , 1.20
Zhang 2024 [50]/Stomach 1.14 1.10 , 1.19
Zhou 2024 [41] /Colorectal adenoma 1.14 1.09 , 1.19
1

Figure S3

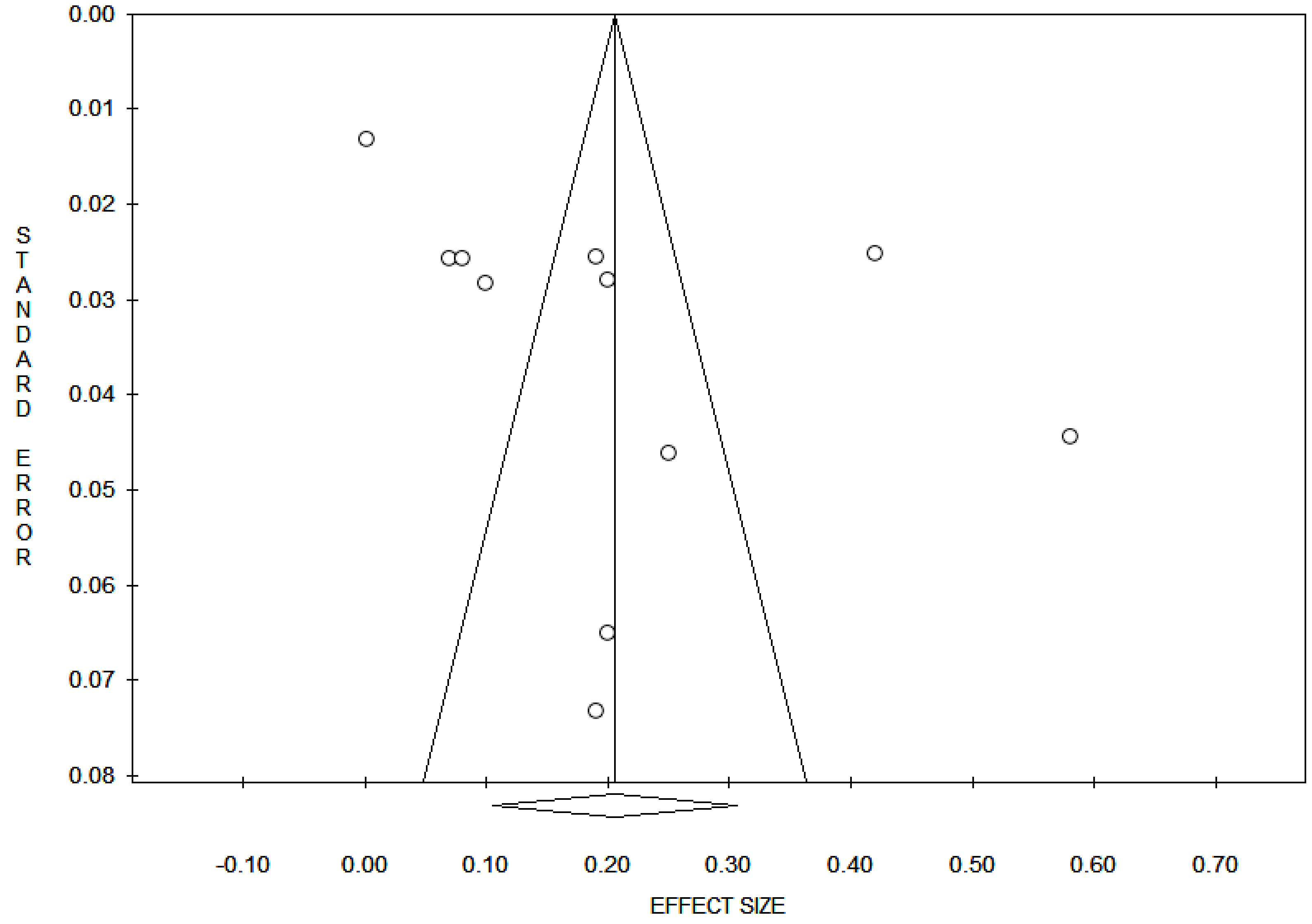
0.00
0.01
0.02
0.03
0.04
0.05
0.06
0.07
0.08
STANDARD ERROR
-0.10
0.00
0.10
0.20
0.30
0.40
0.50
0.60
0.70
EFFECT SIZE

Figure S4

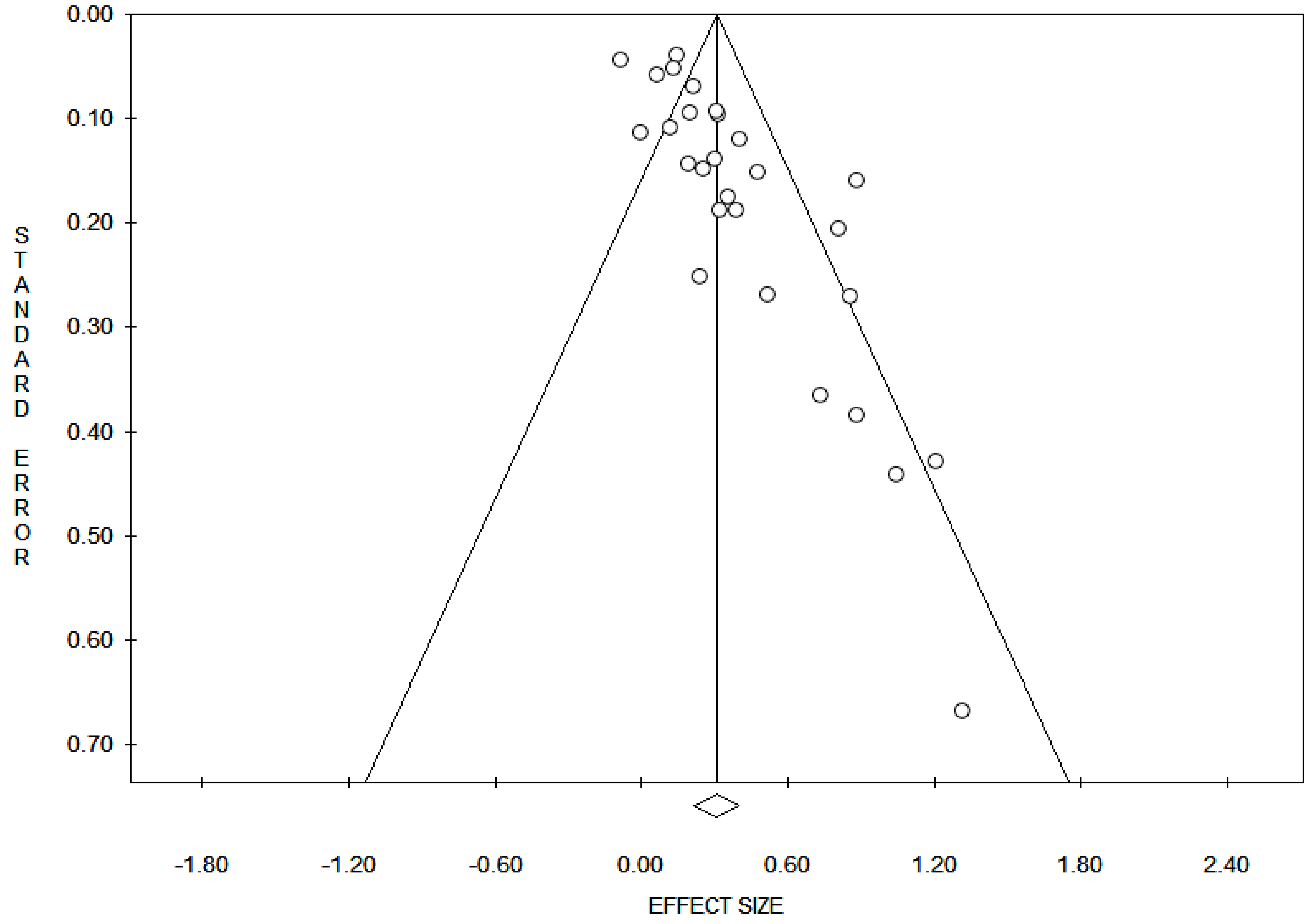

0.00
0.10
0.20
0.30
0.40
0.50
0.60
0.70
STANDARD ERROR
-1.80
-1.20
-0.60
0.00
0.60
1.20
1.80
2.40
EFFECT SIZE

Figure S5

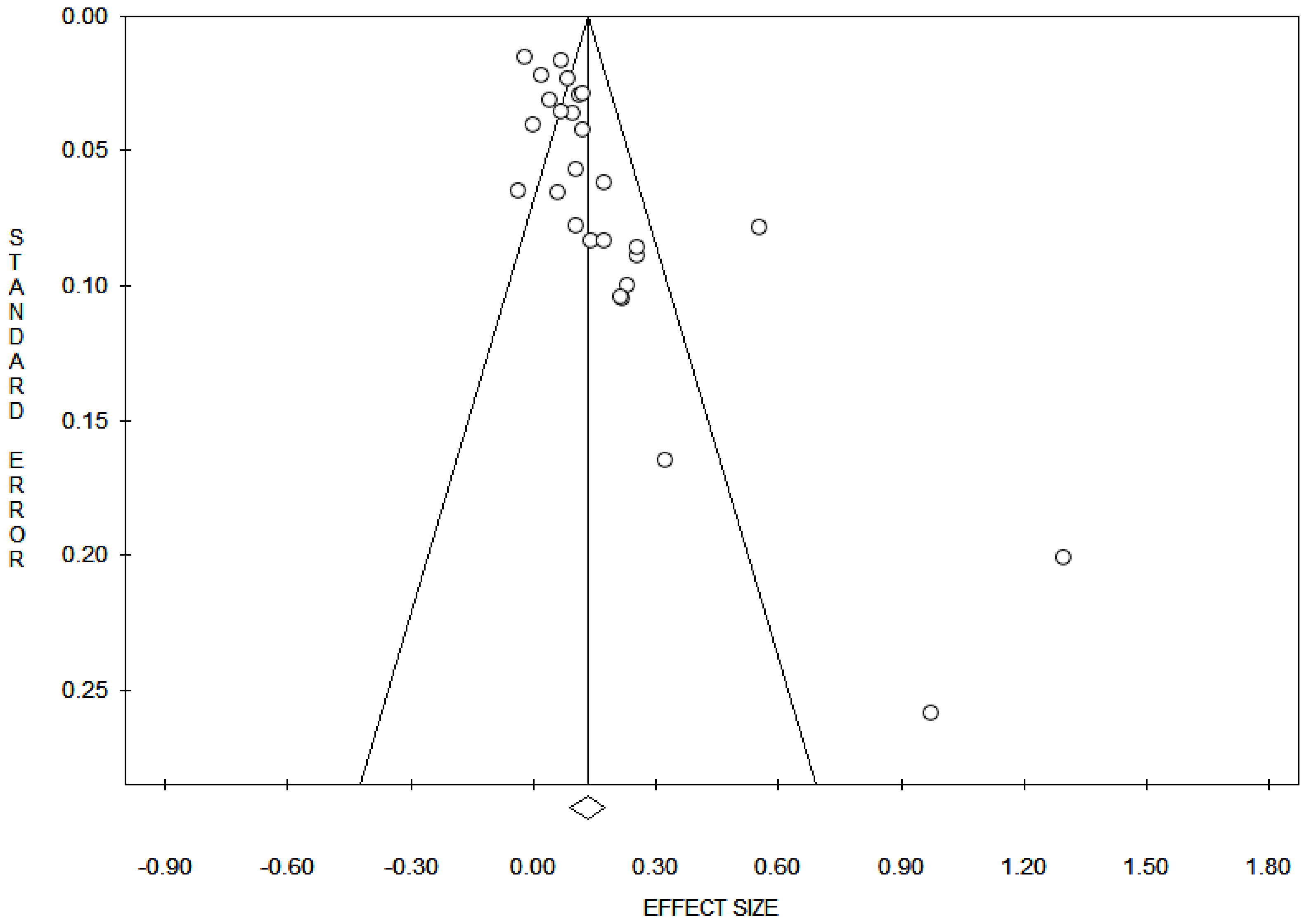
0.00
0.05
0.10
0.15
0.20
0.25
STANDARD ERROR
-0.90
-0.60
-0.30
0.00
0.30
0.60
0.90
1.20
1.50
1.80
EFFECT SIZE

Figure S6
